# Association between markers of glycemia and carotid intima-media thickness: the MARK study

**DOI:** 10.1186/s12872-016-0380-6

**Published:** 2016-10-28

**Authors:** Manuel A. Gomez-Marcos, Leticia Gomez-Sanchez, Maria C. Patino-Alonso, Jose I. Recio-Rodriguez, Natividad Garcia Regalado, Rafel Ramos, Ruth Marti, Cristina Agudo-Conde, Emiliano Rodriguez-Sanchez, Jose A. Maderuelo-Fernandez, Luis Garcia-Ortiz, for the MARK Group

**Affiliations:** 1 Primary Care Research Unit, the Alamedilla Health Center, 37003 Salamanca, Spain; 2 Castilla and León Health Service–SACYL, REDIAPP. IBSAL, Salamanca, Spain; 3 Medicine Department, University of Salamanca, Salamanca, Spain; 4 Statistics Department, University of Salamanca, Salamanca, Spain; 5 CS San Agustín, Primare Care Ibsalut, Palma of Mallorca, Spain; 6 Research Unit, Family Medicine, Girona, Spain; 7 Jordi Gol Institute for Primary Care, Research (IDIAP Jordi Gol), Girona, Spain; 8 Translab Research Group. Department of Medical Sciences, School of Medicine, University of Girona, Girona, Spain; 9 Girona Biomedical Research Institute (IDIBGI), Dr. Trueta University Hospital, Catalonia, Spain; 10 MARK Group. redIAPP: Research Network on Preventive Activities and Health Promotion, Girona, Spain

**Keywords:** Intima-media thickness, Fasting plasma glucose, Postprandial glucose, Glycosylated hemoglobin

## Abstract

**Background:**

Carotid intima-media thickness (C-IMT) is a reliable predictor of cardiovascular events. We Investigated the relationship between markers of glycemia and C-IMT in intermediate-risk cardiovascular patients.

**Methods:**

This study analyzed 427 subjects, aged 35 to 74 years (mean, 60.3 ± 8.5 years), 55 % women, enrolled into the MARK study. Including 231 subjects defined as normal glucose, 104 subjects classified as prediabetes and 92 with type 2 diabetes mellitus. Carotid ultrasound was used to measure C-IMT and the presence of plaques. Fasting plasma glucose (mg/dl) and glycated hemoglobin (%) (HbA1c) were measured using standard enzymatic automated methods. Postprandial glucose (mg/dl) was self-measured by patients at home 2 h after meals (breakfast, lunch and dinner) for 1 day.

**Results:**

The C-IMT shows a positive correlation with fasting plasma glucose, postprandial glucose and HbA1c. Multiple linear regression analysis showed a positive association between HbA1c and C-IMT, with a 0.016 mm and 0.019 mm increase in mean and maximum C-IMT per 1 % increase in HbA1c. In addition, an association between fasting plasma glucose and C-IMT was found with an increase of 0.004 and 0.005 mm in mean and maximum C-IMT per 10 mg/dl in fasting plasma glucose. We also observed a graded association between fasting plasma glucose, postprandial glucose and HbA1c and the presence of carotid target organ damage (TOD), with an odds ratio of 1.013, 1.010 and 1.425, respectively.

**Conclusion:**

The results of this study suggest that the fasting plasma glucose and HbA1c, but not postprandial glucose, are associated with C-IMT media and maximum. The patients who present with a metabolic glucose alteration have more risk of developing carotid TOD.

**Trial registration:**

ClinicalTrials.gov; Identifier: NCT01428934.

**Electronic supplementary material:**

The online version of this article (doi:10.1186/s12872-016-0380-6) contains supplementary material, which is available to authorized users.

## Background

A positive relationship between the glycosylated hemoglobin (HbA1C), fasting plasma glucose and postprandial glucose with cardiovascular morbidity in diabetes patients has been shown in several studies [1, 2]. In people free of diabetes, this association is not clear, and studies suggest that there is a higher association with HbA1c [1, 3–6] than with others parameters. However, patients with type 2 diabetes and those with glucose intolerance present with a carotid thickness that is 0.13 and 0.04 mm wider than the carotid thickness of the control subjects. These results are similar in different ethnicities and in both sexes [7].

The association between HbA1c, fasting glucose and postprandial glycemia with the carotid intima-media thickness (C-IMT) is different in diabetic patients compared with non-diabetic people [8]. Studies that have analyzed the relationship between HbA1c and intima-media thickness of common carotid artery in non-diabetics are inconclusive. Studies showed a positive association between HbA1c and 1-h post load plasma glucose and C-IMT [2, 3]. However, the Dong-gu study [4] showed that HbA1c level was independently associated with arterial stiffness, but not with carotid atherosclerotic parameters, in the overall population without diabetes. Choi et al. [9] recently showed an association with the presence of cholesterol fatty plaques in the coronary artery walls but not with the C-IMT in Korean diabetic patients. However, there was a positive association in Japanese diabetics with fatty plaques of cholesterol on the coronary artery walls [10].

A positive association between glycemia and C-IMT in the general population has been described in several studies [11, 12]. The association between postprandial glucose and the C-IMT that was analyzed by Kato et al. [13], using the 2-h plasma glucose measurement during an oral glucose tolerance test, was positively and independently associated with C-IMT in Japanese subjects with normal glucose tolerance, which is in agreement with the results published by Andreozzi F et al. [8]. Xing F et al. [6] found a relationship between the HbA1c and the basal glucose level C-IMT in prediabetic patients, but this association did not remain after an adjustment for other cardiovascular risk factors. Tanaka et al. [14] showed that the increase in glycemia one hour after 75 g of glucose overload was the only parameter that was associated with C-IMT, but Hot et al. [15] did not show that association.

Thus, the association of normoglycemic levels with subclinical cardiovascular measures remains controversial and is of particular interest, given that this association would potentially affect a larger proportion of the population.

The association between markers of glycemia and vascular structure in patients with intermediate cardiovascular risk has not been determined. In patients with intermediate cardiovascular risk, it is important to analyze new cardiovascular risk factors and the association between them for personalized risk stratification. Therefore, we investigated the relationship between markers of glycemia and vascular structure based on C-IMT in intermediate-risk cardiovascular patients.

## Methods

### Study design

This study enrolled 427 subjects, who were included at the baseline of the MARK study and who underwent fasting glucose, postprandial glucose and glycosylated hemoglobin (HbA1c) tests, and also carotid ultrasonography. The MARK study is a cross-sectional study to evaluate if ankle-brachial index (ABI) measures of arterial stiffness cardio-ankle vascular index (CAVI), postprandial glucose, glycosylated hemoglobin, self-measured blood pressure and presence of comorbidity are independently associated with the incidence of vascular events, and whether they can improve the predictive capacity of the current risk equations in the intermediate-risk population. The second step will be a 5–10-year follow-up to evaluate cardiovascular morbidity and mortality (NCT01428934) [16].

### Study population

Population aged between 35 and 74 years who have an intermediate cardiovascular risk, defined as coronary risk between 5 and 15 % at 10 years according to the Framingham adapted risk equation [17], vascular mortality risk between 1 and 5 % at 10 years according to the SCORE equation [18] or moderate risk according to the 2013 European Society of Hypertension guidelines for the management of arterial hypertension [19]. This study analyzed 427 subjects enrolled in MARK study. Including 231 subjects defined as normal glucose, 104 subjects classified as prediabetes and 92 with type 2 diabetes mellitus. In the 335 patients without prior diagnosis of diabetes we have adapted the cut-offs of HbA1c, fasting plasma glucose (FPG) and postprandial glucose (PG) according to current American Diabetes Association criteria [20]. We have considered prediabetes if HbA1c between 5.7 and 6.4 or FPG between 100 and 125 mg/dL or PG between 140 and 199 mg/dL and not diagnostic de type 2 diabetes mellitus, and metabolism of glucose normal if HbA1c ≤5.7 or FPG ≤100 mg/dL or PG ≤140 and not diagnostic of type 2 diabetes mellitus or prediabetes.

The exclusion criteria were terminal illness, institutionalization at the appointment time or a personal history of atherosclerotic disease. Sample selection was done using a random sample from the population aged 35 to 74 years (both included) who had an intermediate cardiovascular risk. Recruitment and data collection for the study occurred from July 2011 to June 2013. A sample-size calculation indicated that the 427 patients included in this study constituted a sufficient sample for detecting a correlation coefficient of 0.135 between fasting glucose and C-IMT in a two sided test, with a level of significance of 95 % (alfa risk 0.05) and a power of 80 % (beta risk 0.20). The study was approved by an independent ethics committee in the Salamanca health area (Spain), and all participants gave written informed consent, according to the general recommendations of the Declaration of Helsinki [21].

### Measurements

A detailed description has been published elsewhere regarding collection of clinical data, anthropometric measurements and analytical parameters [16].

#### Laboratory determinations

Venous blood sampling was performed between 08:00 and 09:00 after the individuals had fasted and abstained from smoking and the consumption of alcohol and caffeinated beverages for the previous 12 h. Fasting plasma glucose, HbA1c, serum total cholesterol and high-density lipoprotein (HDL) cholesterol concentrations were measured using standard enzymatic automated methods. Blood samples were collected in the Alamedilla Health Center and analyzed at the Salamanca Hospital, which was approved by the Spanish Society of Clinical Chemistry and Molecular Pathology external quality assurance programs. Postprandial glucose (mg/dl) was self-measured by patients at home 2 h after meals (breakfast, lunch and dinner) for 1 day using an Accu-chek® glucometer (Roche Diagnostics Corporation, Spain). Postprandial glucose was calculated as the average of the three measurements. We considered glucose metabolism to be impaired if the patient had diabetes or impaired fasting glucose (between 100 and 126 mg/dl) or HbA1c (between 5.7 and 6.4) or postprandial glucose (between 140 and 200 mg/dl) [14].

#### Office blood pressure

Office blood pressure (BP) was calculated as the average of the last two of three measurements of systolic blood pressure (SBP) and diastolic blood pressure (DBP) made using a validated sphygmomanometer (OMRON Model M10-IT). Measurements were made using the participant’s dominant arm when they were in a seated position after at least 5 min of rest, with a cuff of appropriate size as determined by measurement of the upper-arm circumference according to the European Society of Hypertension recommendations [22].

#### Assessment of vascular structure using carotid intima media thickness

Two trained investigators performed the carotid ultrasound to assess carotid C-IMT before starting the study. Before the study, the reliability of the recordings was evaluated using the intra-class correlation coefficient, which showed values of 0.97 (95 % CI: 0.94 to 0.99) for intra-observer agreement of repeated measurements in 20 subjects, and 0.90 (95 % CI: 0.74 to 0.96) for inter-observer agreement. According to the Bland-Altman analysis, the mean difference for inter-observer agreement (95 % limit of agreement) was 0.01 (−0.03 to 0.06). A Sonosite Micromax ultrasound (SonoSite, EE.UU device paired with a 5–10 MHz multi-frequency high-resolution linear transducer with Sonocal software was used for to automatically measure the C-IMT. There were 120 values obtained automatically, ten measurements in each of the 12 projections, to optimize reproducibility. The common carotid was measured after examining a 10-mm longitudinal section at a distance of 1 cm from the bifurcation, and measurements were also taken of the proximal wall. The lateral (90 °), anterior (45 °) and posterior (135 °) projections in the distal wall followed an axis perpendicular to the artery to distinguish between two lines: one for the intima-blood interface and the other for the media-adventitious interface. A total of six measurements of the right carotid artery and six measurements of the left carotid artery were obtained, and the average values (average mean C-IMT and average maximum C-IMT) were automatically calculated by the software [23]. The measurements were obtained with the subject lying down, with their head extended and slightly turned opposite to the carotid artery that was examined. It was considered as carotid TOD if exists a plaque or a carotid IMT >0.9 mm. Presence of a plaque was identified by an IMT ≥1.5 mm or by a focal increase in thickness of 0.5 mm or 50 % of the surrounding carotid IMT value [19].

#### Others measurements

##### Anthropometric measurements

Body mass index (BMI) was calculated as weight (kg) divided by height squared (m^2^). A value of >30 kg/m^2^ was considered to define obesity.

##### Lifestyles

Tobacco

Smoking history was assessed by asking questions about the participant’s smoking status (smoker/non-smoker) and the number of cigarettes/day for the smokers. We considered smokers to include those who currently smoke or who have stopped smoking within the past year.

Alcohol

Alcohol consumption was assessed through a structured questionnaire and was expressed in grams per week.

Physical activity

Leisure time physical activity (LTPA) practice was collected using the Minnesota LTPA Questionnaire that was validated for Spanish men and women [24, 25]. The questionnaire was administered by trained interviewers and it collected detailed information about physical activity during the preceding year, the number of times this activity was performed and the average duration of each activity on each occasion. Each physical activity has an intensity code, based on the ratio between the metabolic rate during PA practice and the basal metabolic rate. Consumption in mean metabolic equivalents (METs) -min was estimated over 14 days by multiplying the MET in physical activity with the duration (in minutes) and cumulative frequency in the month before the interview.

The individuals performing the different tests were blinded to the patient’s clinical data. All assessments were made within a period of 10 days.

### Statistical analysis

Continuous variables were expressed as the mean ± standard deviation for normally-distributed continuous data, the median (interquartile range, IQR) for asymmetrically-distributed continuous data and as a frequency distribution for categorical data. Statistical normality was tested using the Kolmogorov–Smirnov test. Pearson correlation was performed to analyze the relationship between quantitative variables and Spearman’s correlation was used to analyze the relationship between asymmetrically-distributed continuous data. Analysis of variance (ANOVA) was used to determine differences in the means between more than two categories of quantitative variables for symmetrically distributed continuous data. Comparisons between three or more groups were also made using ANOVA and differences between groups were assessed using the Bonferroni post hoc test.

We performed multiple linear regression analyses, one for each dependent variable, with C-IMT mean and maximum (×100) to facilitate interpretation of fasting glucose, average postprandial glycemia and HbA1c as independent variables. We adjusted by age and gender (Model 1), and by age, gender, heart rate, smoker, body mass index, no HDL-cholesterol, systolic blood pressure, diabetes mellitus, antihypertensive drugs, lipid lowering drugs, antidiabetic drugs, alcohol drinking (gr/week) and METs/min/14 days (Model 2). To improve the final interpretation, the C-IMT was multiplied one hundred-fold. To estimate the odds ratio (OR), two models were constructed using the same adjust as the previous multiple linear regression analyses. The presence of carotid target organ damage (TOD) was considered to be a variable dependent and fasting glucose (mg/dL), average postprandial glycemia (mg/dL), HbA1c (%) and altered glucose metabolism were considered to be independent variables. The data were analyzed using the Statistical Package for the Social Sciences version 20.0 (SPSS, Chicago, IL, USA). A value of *p* < 0.05 was considered statistically significant.

## Results

The characteristics of the study subjects, global diabetics and non-diabetics are shown in Table 1. The mean age was 60.3 ± 8.5 years, and 55.3 % of the subjects were women. The mean C-IMT was 0.738 mm (0.768 ± 0.111 in diabetics and 0.729 ± 0.089 in non diabetics).

**Table 1:** Baseline demographic and clinical characteristics of patients

	Global (*n* = 427)	Diabetics (*n* = 92)	Non-Diabetics (*n* = 335)	*p* value
Age (years)	60.3 ± 8.5	59.7 ± 84.5	81.3 ± 113.4	0.483
Gender (women) n (%)	236 (55.3)	63 (68.5)	173 (51.6)	0.004
Smoker n (%)	82 (21.5)	19 (20.7)	73 (21.8)	0.887
Alcohol drinking (gr/week)	76.6 ± 108.1	60.9 ± 8.8	60.2 ± 8.4	0.089
METs/hour/week	3623 ± 3431	3378 ± 3153	3691 ± 3506	0.440
Body mass index (kg/m^2^)	28.3 ± 4.2	29.8 ± 5.0	27.9 ± 3.9	<0.001
Abdominal perimeter (cm)	97.5 ± 10.6	102.4 ± 11.7	96.2 ± 9.9	<0.001
Obesity patients n (%)	128 (27.6)	38 (41.3)	80 (23.9)	0.001
Office SBP (mmHg)	134 ± 17	137.0 ± 14.7	133.4 ± 17.1	0.067
Office DBP (mmHg)	81 ± 11	79.7 ± 10.1	81.5 ± 11.1	0.169
Office MBP (mmHg)	93 ± 10	93.4 ± 9.2	93.1 ± 9.7	0.783
Office Heart rate (bpm)	70 ± 11	73.0 ± 12.1	69.3 ± 10.8	0.005
Hypertensive patients n (%)	340 (79.6)	76 (82.6)	264 (78.8)	0.468
Antihypertensive Drugs n (%)	230 (53.9)	60 (65.2)	170 (50.7)	0.018
FPG (mg/dL)	97.9 ± 31.1	137.5 ± 46.1	87.0 ± 10.1	<0.001
Average PG (mg/dL)	120.2 ± 32.4	152.4 ± 9.5	111.3 ± 17.6	<0.001
HbA1c (%)	5.9 ± 1.0	7.2 ± 1.4	5.6 ± 0.3	<0.001
Diabetics patients n (%)	92 (21.5)	92 (21.5)	0 (0.0)	---
Antidiabetic drugs n (%)	76 (17.8)	76 (82.6)	0 (0.0)	---
Total Cholesterol (mg/dl)	215.0 ± 38.93	193.1 ± 41.1	221.0 ± 36.1	<0.001
LDL Cholesterol (mg/dl)	134.3 ± 34.5	113.4 ± 35.5	140.1 ± 32.0	<0.001
HDL-Cholesterol (mg/dl)	55.1 ± 14.3	51.6 ± 13.0	56.1 ± 14.5	0.009
Tryglicerides (mg/dl)	129.4 ± 92.6	149.0 ± 138.4	124.0 ± 74.8	0.022
No HDL-Cholesterol (mg/dl)	80.2 ± 13.8	79.8 ± 16.3	80.2 ± 13.0	0.805
Dyslipidemia patients n (%)	363 (85)	66 (71.7)	297 (88.7)	<0.001
Lipid lowering drugs n (%)	158 (37)	56 (60.9)	102 (30.4)	<0.001
Average mean IMT (mm)	0.738 ± 0.096	0.768 ± 0.111	0.729 ± 0.089	0.001
Carotid plaques n (%)	75 (17.6)	28 (30.4)	47 (14.1)	<0.001
TOD carotid n (%)	77 (18.0)	29 (31.5)	48 (14.3)	<0.001
Average maxima IMT (mm)	0.906 ± 0.117	0.942 ± 0.136	0.896 ± 0.109	0.001

Values are means and standard deviations (SD) for distributed continuous data, and absolute frequencies and percentages (%) for categorical data

*METs* metabolic equivalents, *SBP* systolic blood pressure, *DBP* diastolic blood pressure, *MBP* mean blood pressure, *FPG* fasting plasma glucose, *PG* postprandial glucose, *HbA1c* glycosylated hemoglobin, *LDL* low density lipoprotein, *HDL* high density lipoprotein, *IMT* intima-media thickness of common carotid artery, *TOD* target organ damage

No HDL-Cholesterol mg/dl = (Total Cholesterol – HDL Cholesterol)

TOD = Plaque or carotid IMT >0.90 mm

**Fig. 1:**
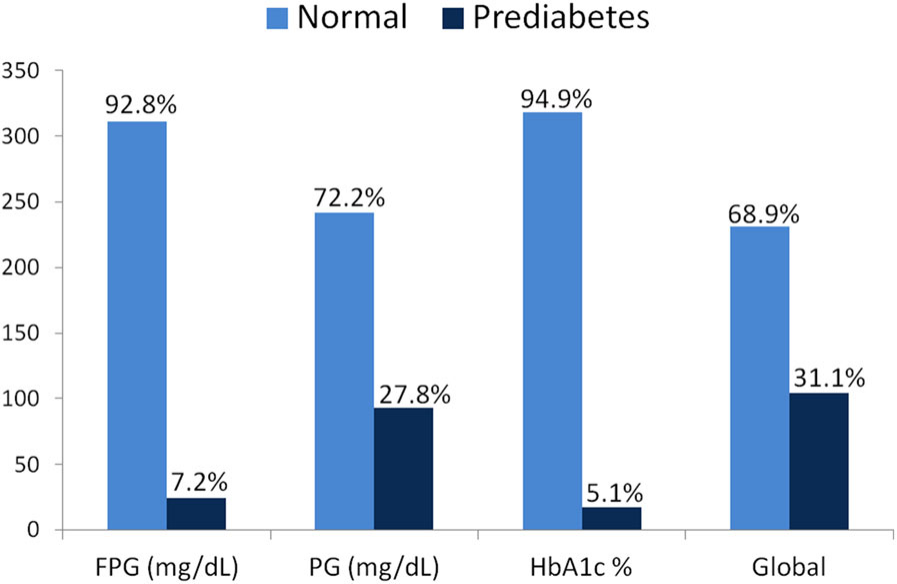
Percentage of subjects depending on the test used in the diagnosis of normal glucose metabolism and prediabetes, FPG fasting plasma glucose. PG postprandial glucose. HbA1c glycosylated hemoglobin

Among the 335 non-diabetic subjects, 231 had normal glucose metabolism and 104 prediabetes, the percentage of subjects depending on the test used in the diagnosis shown in Fig. 1.

C-IMT shows a positive relationship with fasting glucose, average postprandial glycemia and HbA1c (Table 2). The 196 patients who have a glucose metabolic alteration also show mean C-IMT and C-IMT values of 0.039 and 0.044, which are higher than those in subjects without any glucose metabolic alteration (Additional file 1: Table S1).

**Table 2:** Bivariate correlations of markers of dysglycemia with IMT and other cardiovascular risk factors

	FPG (mg/dL)	Average PG (mg/dL)	HbA1c %
Average mean IMT (mm)	0.179**	0.182**	0.191**
Maxima men IMT (mm)	0.173**	0.207**	0.182**
Age (year)	0.058	0.157*	0.019
Body mass index	0.201**	0.081	0.226**
Heart rate. bpm	0.184**	0.123*	0.155**
No HDL-Cholesterol (mg/dl)	0.171**	0.069	0.065
Systolic Blood Pressure (mm Hg)	0.115*	0.049	0.059
Alcohol drinking (gr/week)	−0.064	−0.075	−0.094
METs/hour/week	−0.130*	−0.109	−0.116

*IMT* intima-media thickness of common carotid artery, *FPG* fasting plasma glucose, *PG* postprandial glucose, *HbA1c* glycosylated hemoglobin, *HDL* high density lipoprotein, *METs* metabolic equivalents

*P*-values by Pearson correlation. *p < 0.05 **p < 0.01

**Fig. 2:**
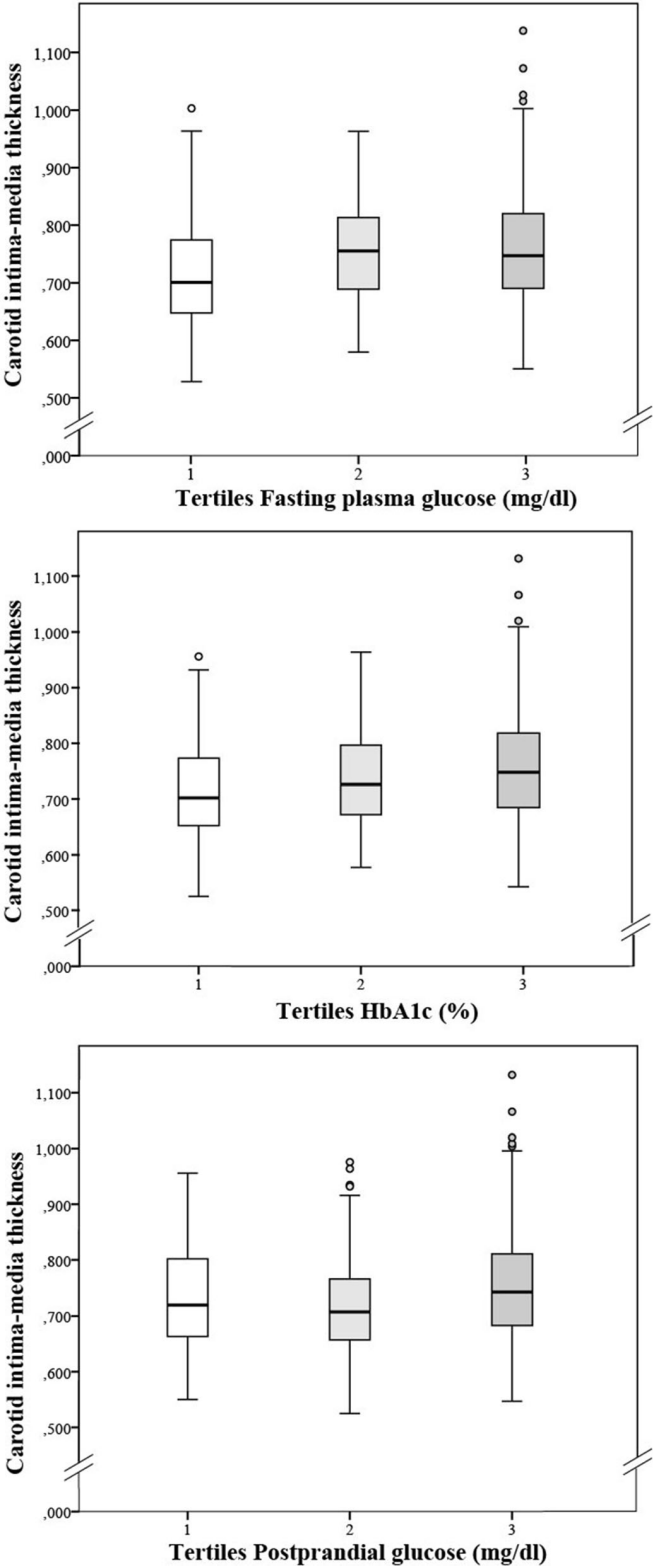
ANOVA test results: Carotid intima media thickness by tertiles of fasting plasma glucose (mg/dl), postprandial glucose (mg/dl) and glycated hemoglobin (%)

Figure 2 and Additional file 1: Table S2 show the values of the C-IMT organized by tertiles of fasting glucose (mg/dL), average postprandial glycemia (mg/dL) and HbA1c.

A multiple linear regression analysis was performed (Table 3). In model 1, the C-IMT is associated with different parameters that were used to measure glucose. After fully adjusting for all parameters (Model 2), C-IMT maintains a positive association with fasting glucose and with HbA1c. For each 1 % increase in HbA1c, the C-IMT mean increased 0.016 mm and the C-IMT maximum increased 0.019 mm. For each 10-mg increase in fasting plasma glucose, the C-IMT mean and maximum increase 0.004 and 0.005 mm, respectively.

**Table 3:** Multiple regression analysis with vascular structure as dependent variable and markers of dysglycemia as independent variable

Dependent variable:	β	CI 95 %	*p* value
Average mean IMT Model 1			
FPG (mg/dL)	0.046	0.020 to 0.073	0.001
Average PG (mg/dL)	0.035	0.009 to 0.060	0.008
HbA1c (%)	1.658	0.813 to 2.503	<0.001
Average maxima IMT Model 1			
FPG (mg/dL)	0.055	0.022 to 0.088	0.001
Average PG (mg/dL)	0.041	0.009 to 0.072	0.012
HbA1c %	1.955	0.908 to 0.302	<0.001
Average mean IMT Model 2			
FPG (mg/dL)	0.042	0.002 to 0.082	0.040
Average PG (mg/dL)	0.021	−0.010 to 0.051	0.188
HbA1c (%)	1.589	0.435 to 2.771	0.009
Average maxima IMT Model 2			
FPG (mg/dL)	0.052	0.002 to 0.102	0.042
Average PG (mg/dL)	0.024	−0.015 to 0.062	0.225
HbA1c (%)	1.907	0.435 to 3.378	0.011

Dependent variable: IMT: Intima-media thickness of common carotid artery

Indepedent variable: fasting glucose (mg/dL), average postprandial glycemia (mg/dL) and HbA1c (%)

Adjusted by: Model 1: age and gender

Modelo 2: age, gender, heart rate, smoker, body mass index, no HDL-Cholesterol, systolic blood pressure, diabetes mellitus, antihypertensive drugs, lipid lowering drugs, antidiabetic drugs alcohol drinking (gr/week), METs/hour/week

*β* regression coefficient, *CI* confidence interval, *IMT* intima-media thickness of common carotid artery, *FPG* fasting plasma glucose, *PG* postprandial glucose, *HbA1c* glycosylated hemoglobin

The association between IMT average mean and glucose in diabetic subjects and nondiabetic are set out in the Additional file 1: Table S3.

Table 4 shows the OR of the different markers of glycemia for the presence of carotid TOD. In Model 2, the association between fasting plasma glucose, postprandial glucose and HbA1c with the presence of carotid TOD is maintained (OR = 1.013, OR = 1.010 and OR = 1.425, respectively). Patients who have a glucose metabolic alteration have twice the risk of presenting with a carotid TOD (OR = 1.920).

**Table 4:** Logistic regression analysis with vascular structure as dependent variable and markers of dysglycemia as independent variable

Dependent variable:	OR	CI 95 %	*p* value
With and without TOD of cartic Modelo 1			
FPG (mg/dL)	1.012	1.005 to 1.020	0.001
Average PG (mg/dL)	1.012	1.005 to 1.020	0.001
HbA1c (%)	1.492	1.189 to 1.872	0.001
Altered glucose metabolism	2.153	1.217 to 3.629	0.004
With and without TOD of carotic Modelo 2			
FPG (mg/dL)	1.013	1.001 to 1.025	0.039
Average PG (mg/dL)	1.010	1.001 to 1.019	0.010
HbA1c (%)	1.425	1.026 to 1.980	0.034
Altered glucose metabolism*	1.920	1.079 to 3.415	0.026

Dependent variable: target organ damage of carotid

Indepedent variable: fasting glucose (mg/dL), average postprandial glycemia (mg/dL) and HbA1c (%)

Adjusted by: Model 1: age and gender

Modelo 2: age, gender, heart rate, smoker, body mass index, no HDL-Cholesterol, systolic blood pressure, diabetes mellitus, antihypertensive drugs, lipid lowering drugs, antidiabetic drugs alcohol drinking (gr/week), METs/hour/week

*OR* odds ratio, *CI* confidence interval, *TOD* target organ damage, *FPG* fasting plasma glucose, *PG* postprandial glucose, *HbA1c* glycosylated hemoglobin

TOD = Plaque or carotid IMT >0.90 mm (*n* = 77)

*Altered glucose metabolism including diabetes and prediabetes subjects (*n* = 196)

The 77 patients who presented with carotid TOD also presented with fasting glucose levels that were 15.10 mg/ml higher, average postprandial glycemia that was 18.69 mg/ml higher and HbA1c that was 0.47 % higher than those without carotid TOD.

## Discussion

The results of this study suggest that, in patients with an intermediate cardiovascular risk, there is a positive association between C-IMT and the basal glycemia and HbA1c levels, which is independent of age, drug treatment and other confounding factors. The probability of developing carotid TOD increases with different glycemia parameters. Similarly, patients who present with a glucose metabolic alteration have twice the risk of developing TOD.

In this study, for each percentage point increase in HbA1c, the mean C-IMT increases by 0.016 mm and the maximum C-IMT increases by 0.019. Our results are consistent with previous studies that were performed in the Chinese general population [26], and also consistent with results from the non-diabetic population of a multi-ethnic atherosclerosis study [11] However, Haring et al. [27] found a positive association between HbA1c and mean C-IMT, with a 0.02 mm increase in C-IMT per 1 % increase in HbA1, but this association did not remain in the longitudinal analysis.

The association with 2 h postprandial glycemia was lost after adjusting for confounding factors, and the logistic regression maintained an OR of 0.010. Previous publications had different conclusions. For example, 1 h after glucose overload glycemia was found to be the only factor associated with C-IMT after adjusting for different cardiovascular risk factors in a linear regression analysis [14]. This association was not present 2 h after glucose overload, but the latter association was found by others authors [6, 13]. Similarly, in type 2 diabetes patients, the maximum increase in glycemia is registered within the first hour after a meal, and this showed a stronger correlation with C-IMT than with other glycemia markers such as HbA1C, fasting glucose and glucose 2 h after a meal [28].

A glucose metabolic alteration doubles the risk of developing carotid TOD. These results are similar to those of Kurihara et al. [29], who concluded that arteriosclerotic disease in the coronary artery and the vulnerability to plaque are more progressed in pre-diabetics and diabetics than in non-diabetics. However, the relationship in pre-diabetic patients is statistically significant after adjustment for other cardiovascular risk factors [6].

Other studies that also analyzed the association between different measurements of glycemia using C-IMT [1, 13, 30, 31] and found divergent results. In our study, the independent association of the HbA1C and fasting glucose with the mean and maximum C-IMT remains after the adjustment for age, sex, drug treatment and others cofounding cardiovascular factors, including the association between the above-mentioned parameters and glucose levels 2 h after a meal. These differences could be explained by the heterogeneity of the studies, different protocols used to measure C-IMT, different parameters used to assess glycemia, different ethnicities included in the studies (for example, Caucasian vs. Asian) and other features of the subjects such as body mass index or the prevalence of different cardiovascular risk factors and different variables used to adjust for confounding factors.

These studies suggest that more prospective studies that analyze the relationship between glycemia and the C-IMT are needed, and that they should include different ethnicities. Studies that analyze how glycemia at 1 and 2 h after a meal or glucose overload develops, and its association with C-IMT are required to establish which postprandial glycemia value is better associated with subclinical atherosclerotic disease.

### Limitations

This study has several limitations that should be considered in the interpretation of our results. First, because the study sample comprised exclusively Caucasian adults with an intermediate cardiovascular, our results may not be generalizable to other ethnic groups. The observational study design and the cross-sectional nature of the positive association between markers of glycemia and C-IMT as an intermediate risk marker for subclinical atherosclerosis does not prove causality. Finally, glucose intolerance was not performed with oral glucose tolerance test with 75 g of glucose. It was determined with the mean capillary blood glucose 2 h after breakfast, lunch and dinner. However, this is the first study to examine the relationship between the markers of glycemia and vascular structure, as assessed using the C-IMT, in intermediate-risk cardiovascular patients.

## Conclusion

In conclusion, the results of this study suggest that the fasting plasma glucose and the HbA1c levels, but not postprandial glucose, are associated with the mean and maximum C-IMT. Patients who present the glucose metabolic alteration have more risk of developing carotid TOD compared with patients with no glucose metabolic alterations.

## Additional file

Additional file 1: Table S1. Parameters of vascular structure by tertiles of markers of glycemia. (DOCX 17 kb)

## Abbreviations

ABI: Ankle-brachial index
BMI: Body mass index
BP: Blood pressure
CAVI: Cardio-ankle vascular index
C-IMT: Carotid intima-media thickness
cSBP: Central systolic blood pressure
DBP: Diastolic blood pressure
HbA1c: Hemoglobin A1c
HDL: High-density lipoprotein
IQR: Interquartile range
LTPA: Leisure time physical activity
METs: Metabolic equivalents
PAD: Peripheral arterial disease
SBP: Systolic blood pressure
TOD: Target organ damage
