# Supplementary material for: Association between markers of glycemia and carotid intima-media thickness: the MARK study
Source: BMC Cardiovasc Disord. 2016 Oct 28;16:203. doi: 10.1186/s12872-016-0380-6 (PMC5084362; doi:10.1186/s12872-016-0380-6)
Supplement: Additional file 1: Table S1. — Parameters of vascular structure by tertiles of markers of glycemia. (DOCX 17 kb) [file 12872_2016_380_MOESM1_ESM.docx]

**TABLE S1: Values of IMT in patients with and without altered glucose metabolism.**

|  | **Yes (n=196)** | **No (n=231)** | **Differences** | **p** |
| --- | --- | --- | --- | --- |
| **Average mean IMT (mm)** | 0.759±*0.104* | 0.720±0.088 | 0.039 | <0.001 |
| **Average maxima IMT (mm)** | 0.930±0.128 | 0.886±0.108 | 0.044 | <0.001 |

Values are means (standard deviations (SD).

Abbreviations: IMT cardio-ankle vascular index.

Altered glucose metabolism including diabetes and prediabetes subjects.

**Table 2S: Parameters of vascular structure by tertiles of markers of glycemia.**

|  | **1^st^tertil** | **2^nd^tertil** | **3^rd^tertil** | | **p** | |  |  |
| --- | --- | --- | --- | --- | --- | --- | --- | --- |
| **FPG (mg/dL)** | (n=136; < 83.56) | (n=138; 83.56-94.10) | | (n=153; >94.10) | |  | |  |
| Mean IMT ^*†^ | 0.714±0.092 | 0.744±0.086 | 0.753±0.103 | | **0.002** | |  |  |
| Maxima IMT ^*†^ | 0.879±0.113 | 0.913±0.107 | 0.925±0.126 | | **0.003** | |  |  |
|  |  |  |  | |  | |  |  |
| **PG (mg/dL)** | (n=142; <104.90) | (n=141; 105.00-121.30) | (n=144; >121.31) | | |  | | |
| Mean IMT ^†‡^ | 0.732±0.090 | 0.724±0.092 | 0.756±0.102 | | **0.013** | |  |  |
| Maxima IMT ^†‡^ | 0.899±0.108 | 0.892±0.115 | 0.927±0.126 | | **0.027** | |  |  |
|  |  |  |  | |  | |  |  |
| **HbA1c %** | (n=147; <5.50) | (n=140; 5.51-5.80) | (n=140; >5.81) | |  | |  |  |
| Mean IMT ^†‡^ | 0.716±0.086 | 0.736±0.089 | 0.762±0.106 | | **<0.001** | |  |  |
| Maxima IMT ^†‡^ | 0.883±0.104 | 0.903±0.112 | 0.933±0.130 | | **0.001** | |  |  |
|  |  |  |  | |  | |  |  |

Values are means (standard deviations (SD).

IMT cardio-ankle vascular index. FPG fasting plasma glucose. PG postprandial glucose. HbA1c glycosylated hemoglobin.

TOD: target organ damage. TOD carotid: IMT > 0.90 mm or plaques with a diameter of 1.5 mm or a focal increase of 0.5 mm or 50% of the adjacent IMT

* p<0.05 between 1^st^ and 2^nd^ tertil; † p <0.05 between 1^st^ and 3^rd^ tertil; ‡ p<0.05 between 2^st^ and 3^th^ tertil.

*p by ANOVA using Bonferroni post hoc test.

**Table 3 S:** **Multiple regression analysis with vascular structure as dependent variable and markers of glycemia as independent variable in diabetics and non-diabetics.**

|  | **Diabetics (n=92)** | | | | **Non-diabetics (n=335)** | | |
| --- | --- | --- | --- | --- | --- | --- | --- |
| **Dependent variable** | | β | CI 95% | p | β | CI 95% | p |
| **Mean IMT Model 1** | |  |  |  |  |  |  |
| FPG (mg/dL) | | 0.040 | -0.007 to 0.086 | 0.093 | 0.036 | -0.050 to 0.123 | 0.407 |
| Average PG (mg/dL) | | 0.025 | -0.019 to 0.068 | 0.262 | 0.014 | -0.036 to 0.064 | 0.584 |
| HbA1c (%) | | 1.265 | -0.226 to 2.756 | 0.095 | 3.104 | 0.320 to 5.889 | 0.029 |
| **Mean IMT Model 2** | |  |  |  |  |  |  |
| FPG (mg/dL) | | 1.691 | -4.748 to 8.130 | 0.603 | -0.022 | -0.107 to 0.063 | 0.611 |
| Average PG (mg/dL) | | 1.031 | -5.637 to 7.699 | 0.759 | 0.001 | -0.046 to 0.049 | 0.959 |
| HbA1c (%) | | 1.613 | -0.134 to 3.360 | 0.070 | 1.670 | -1.044 to 4.384 | 0.227 |

**Dependent variable:** IMT*100: Intima-media thickness of common carotid artery.

**Indepedent variable:** fasting glucose (mg/dL), average postprandial glycemia (mg/dL) and HbA1c (%).

**Adjusted by:** Model 1: age and gender.

Model 2: age, gender, heart rate, smoker, body mass index, no HDL-Cholesterol, systolic blood pressure, diabetes mellitus, antihypertensive drugs, lipid lowering drugs, antidiabetic drugs alcohol drinking (gr/week), METs/min/14 days.

Abbreviations: CI conﬁdence interval. IMT cardio-ankle vascular index. FPG fasting plasma glucose. PG postprandial glucose. HbA1c glycosylated hemoglobin.
